# A Way to Get a Diploma

**Published:** 1846-11

**Authors:** 


"*A way to get a Diploma.*—There is a certain advertising quack in this city, who is said to have obtained a diploma from a Medical College, in the following manner. When he first came to this country he commenced peddling about the streets with a small basket on his arm, which barely enabled him to support his family; but meeting with one of his countrymen, an old German physician, who had been well educated, and had enjoyed a good practice, until he became dissipated, which brought upon him disgrace and degradation, and prepared him for baser deeds and meaner associations, said physician, on coming to this country and becoming acquainted with our hero of the toy-basket, and his peculiar mind, advised him to become a doctor. How can I do that when I have no previous education? asked the pedlar. The German doctor replied, You must feign that you cannot speak English, and I will interpret for you, but we must not let the Professors know that I am a Physician. All preliminary arrangements being made, and the story having been given out, that the Dutch pedlar had been a surgeon in Napoleon's army, etc., he was examined, and his friend interpreted to suit the case.

The result was, *that he passed a pretty fair examination considering his having been educated in another country, and being wholly unacquainted with our language and mode of education.*

The diploma being obtained, served him as a passport into the Medical Society of the city and county of New-York.

We next find his flaring advertisements in nearly all of our city papers, telling of his *celebrated elixirs and wonderful cures.*

This is but one of the impostors, who have crept into the once highly honorable society, composed of the medical profession of this city, but now, alas! how changed.—*N. Y. Med. and Surg. Reporter.*

